# Current Progress on Predictive Biomarkers for Response to Immune Checkpoint Inhibitors in Gastric Cancer: How to Maximize the Immunotherapeutic Benefit?

**DOI:** 10.3390/cancers15082273

**Published:** 2023-04-13

**Authors:** Yongqing Liu, Pengbo Hu, Liang Xu, Xiuyuan Zhang, Zhou Li, Yiming Li, Hong Qiu

**Affiliations:** Department of Oncology, Tongji Medical College, Tongji Hospital, Huazhong University of Science and Technology, Wuhan 430030, China; m202276286@hust.edu.cn (Y.L.);

**Keywords:** gastric cancer, immunotherapy, immune checkpoint inhibitors, biomarkers

## Abstract

**Simple Summary:**

The landscape of gastric cancer treatment has changed owing to the widespread use of immune checkpoint inhibitors. Only a subset of patients, though, have reaped substantial benefits. One of the crucial issues in current research is seeking efficacious biomarkers to identify favorable populations for immunotherapy. In this review, we present a summary of the predictive biomarkers that have lately been exploited or investigated in clinical studies.

**Abstract:**

Gastric cancer is the fifth most prevalent cancer and the fourth leading cause of cancer death globally. Delayed diagnosis and pronounced histological and molecular variations increase the complexity and challenge of treatment. Pharmacotherapy, which for a long time was systemic chemotherapy based on 5-fluorouracil, is the mainstay of management for advanced gastric cancer. Trastuzumab and programmed cell death 1 (PD-1) inhibitors have altered the therapeutic landscape, contributing to noticeably prolonged survivorship in patients with metastatic gastric cancer. However, research has revealed that immunotherapy is only beneficial to some individuals. Biomarkers, such as programmed cell death ligand 1 (PD-L1), microsatellite instability (MSI), and tumor mutational load (TMB), have been shown to correlate with immune efficacy in numerous studies and are increasingly employed for the selection of patients most likely to respond to immunotherapy. Gut microorganisms, genetic mutations like POLE/POLD1 and NOTCH4, tumor lymphoid infiltrating cells (TILs), and other novel biomarkers have the potential to develop into new predictors. Prospective immunotherapy for gastric cancer should be guided by a biomarker-driven precision management paradigm, and multidimensional or dynamic marker testing could be the way to go.

## 1. Introduction

In terms of incidence and mortality, gastric cancer (GC) ranks among the most common cancers worldwide. According to data from GLOBOCAN 2020, there are over 1089 million new cases of gastric cancer each year, coupled with roughly 769,000 deaths worldwide, ranking it as the fifth and fourth most prevalent malignancies, respectively [1]. Chronic Helicobacter pylori infection, smoking, and a harmful diet of excess nitrites are all preventable risk factors for stomach cancer [2,3]. The decrease in the incidence of gastric cancer over the past 50 years can be attributed to improved living conditions and standardized treatment against H. pylori. However, the prognosis of patients with gastric cancer remains unsatisfactory owing to the early symptoms are not obvious, and most patients are at an advanced stage when diagnosed. In China, up to 64.7% of patients are in stage III or IV and even in countries that have universal screening programs, such as Japan, half of the patients are still in the advanced stage of the disease [4].

In general, comprehensive therapeutic strategies are applied to treat gastric cancer. The cornerstone of gastric cancer management nowadays is surgery, especially the increasing popularity of neoadjuvant therapy in recent years has dramatically improved the survival rate of postoperative patients [5,6,7,8,9]. Surgery, however, is not an option for the overwhelming majority of patients with advanced metastatic disease. For a long time, the median overall survival (OS) of advanced gastric cancer managed with conventional first-line chemotherapy regimens (dual or triplet chemotherapy based on platinum or fluorouracil) was merely about 12 months, a short survival period characterized by severe drug toxicity and substantial patient suffering [10,11]. As clinical data accumulate on the benefits of immunotherapy for gastric cancer, authoritative guidelines, such as those issued by the European Society of Medical Oncology (ESMO), National Comprehensive Cancer Network (NCCN), and Chinese Society of Clinical Oncology (CSCO), have added immunotherapy as a recommended treatment option for advanced gastric cancer [12,13,14]. The three primary types of immunotherapy that are currently available comprise immune checkpoint inhibitors (ICIs), cellular immunotherapy, and anti-tumor vaccinations [15]. ICIs, the most common type of immunotherapy at the moment, are generally composed of monoclonal antibodies that target the programmed cell death 1 (PD-1), programmed cell death ligand 1 (PD-L1), and cytotoxic T-lymphocyte associated antigen 4 (CTLA-4) [16,17]. They increase the anti-tumor effect of T cells by suppressing the negative regulatory mechanisms of T cells.

**Figure 1:**
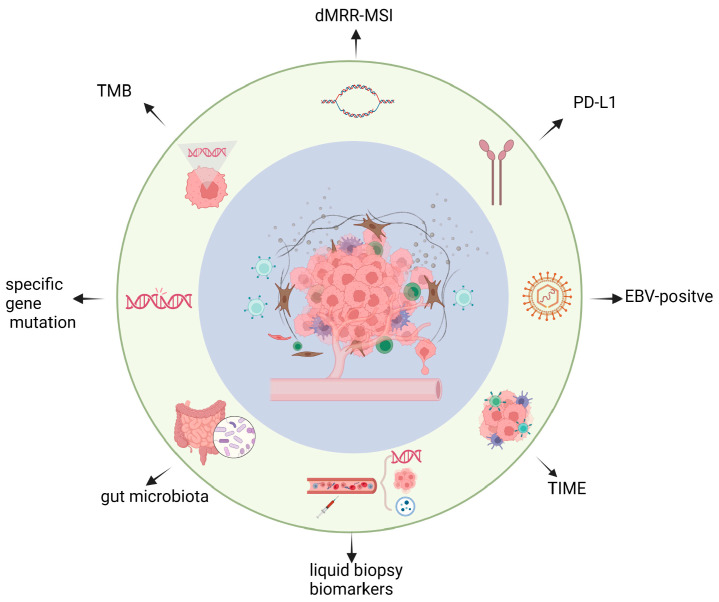
Overview of predictive biomarkers for immune checkpoint inhibitors in gastric cancer. Key elements on predictive biomarker progress for the efficacy of immune checkpoint inhibitors therapy are briefly precented in the figure, including PD-L1, dMRR/MSI, TMB, EBV-positive status, tumor immune microenvironment (TIME), specific genetic mutation, gut microbiota, liquid biopsy biomarkers.

Immunotherapy has transformed the treatment of many advanced gastric cancer patients, but ICIs are frequently ineffective in the majority of patients [18]. In order to identify patients who will truly benefit from ICIs intervention, clinicians and oncologists have been investigating biomarkers that can forecast immunotherapy efficacy. In this review, we will give a brief overview of the clinical research on ICIs for gastric cancer, mainly emphasizing the first-line therapy’s phase III trials. The article’s main focus will be a meticulous review of recent advances in biomarkers, including common biomarkers like PD-L1, dMRR/MSI, TMB, and EBV-positive status, as well as emerging ones like tumor immune microenvironment (TIME), specific genetic mutations, gut microbiota, and liquid biopsy biomarkers (Figure 1).

## 2. Recent Clinical Trials of ICIs in Gastric Cancer

Generally, the immune system in our body can identify and eliminate aberrant cells in time to prevent tumorigenesis. In order to prevent an excessive immune response, simultaneously, the body has physiological immune down-regulation mechanisms that ensure a reasonable limitation of the immune response. Immune checkpoints, which include CTLA-4, PD-1 and its ligand PD-L1, act as inhibitory regulatory molecules that brake the immune system. Nevertheless, tumor cells have the ability to express PD-L1, which binds to PD-1 on the surface of T cells, allowing them to be neglected by the immune surveillance, thus enabling the immune escape [19]. CTLA-4 is a transmembrane protein with high homology to the T cell surface receptor, which interacted competitively with the costimulatory molecule B7 and, thus, inhibited T cell activation. Today’s ICIs products are developed substantially on the basis of the tumor immunosuppressive mechanism described above [20].

### 2.1. Anti-PD-1 Antibody

The monoclonal antibodies that antagonize PD-1 mainly include nivolumab, pembrolizumab, sintilimab, etc. Several representative clinical trials and their findings are listed in Table 1.

Due to the success of the ATTRACTION-02 trial [21], nivolumab has been approved in China as a third-line therapy for people with advanced stomach cancer. Compared with the placebo arm, the nivolumab arm had a significantly longer OS (4.14 vs. 5.26 months). Subsequently, nivolumab commenced entering the first-line therapy studies. Nivolumab plus chemotherapy resulted in a longer PFS of 10.5 months than chemotherapy alone of 8.3 months, according to the phase III ATTRACTION-04 study [22], although there was no statistically significant difference in OS (17.5 vs. 17.2 months, *p* > 0.05). In the phase III Checkmate649 trial [23], nivolumab with chemotherapy is compared to chemotherapy alone as the first-line therapy for advanced gastric cancer. In populations with a PD-L1 CPS ≥ 5, three-year follow-up results showed that the median OS of the combination group was 14.4 months, far more than the 11.1 months in the single-agent group (HR 0.70, 95%CI 0.61–0.81). Meanwhile, the PFS benefited from 6.1 months in the monotherapy group to 8.3 months in the combination group (HR 0.70, 95%CI 0.60–0.81). A similar outcome occurred in all randomized individuals. These results reaffirmed that the routine first-line therapy for individuals with advanced gastric cancer could involve the addition of nivolumab to the chemotherapy [30].

Pembrolizumab is another monoclonal antibody widely used in clinics. As second-line therapy for advanced gastric cancer, pembrolizumab failed to significantly outperform paclitaxel in terms of overall survival in KEYNOTE-061 (mOS: 9.1 vs. 8.3 months; mPFS:1.5 vs. 4.1 months) [25]. The most recent KEYNOTE-859 study further cemented pembrolizumab’s position as the first-line treatment for advanced gastric cancer, even though the phase III KEYNOTE-062 trial found that it failed to significantly prolong OS compared to chemotherapy alone [26,27,31]. The median OS was 12.9 months of pembrolizumab plus FP or CAPOX group versus 11.5 months of placebo in combination with chemotherapy. Pembrolizumab noticeably pronged median PFS from 5.6 months to 6.9 months, objective remission rate (ORR) from 42.0% to 51.3%, and duration of remission (DOR) from 5.7 months to 8 months. The effectiveness and safety of pembrolizumab combined with trastuzumab and chemotherapy in the first-line therapy of HER-2 positive, unresectable or metastatic gastric cancer and esophageal junction adenocarcinoma were assessed in another phase III KEYNOTE-811 research [28]. The ORR was 74.4% in the pembrolizumab arm, an improvement of 22.7% over the placebo arm, according to the interim analysis. Pembrolizumab, which combines trastuzumab with chemotherapy drugs like fluoropyrimidine or platinum in the first-line treatment of locally advanced, unresectable, or metastatic HER-2-positive gastric cancer, has been expedited approved by the U.S. Food and Drug Administration (FDA) depended on the findings of this study.

Sintilimab is a recombinant fully-humanized monoclonal antibody originating from China. In phase 3, ORIENT-16 research [29], a significant reduction in the risk of death was observed in the sintilimab plus chemotherapy group for patients either with a PD-L1 CPS ≥ 5 (HR 0.66, 95%CI 0.505–0.864) or overall in the randomized population (HR 0.766, 95%CI 0.626–0.936). In patients with CPS ≥ 5 and all randomized individuals, the median OS of the sintilimab group was revealed to have a survival benefit over placebo (18.4 vs. 12.9 months).

### 2.2. Anti-PD-L1 Antibody

Avelumab, an anti-human PD-L1 monoclonal antibody, has demonstrated good clinical activity and safety as first-line maintenance or second-line therapy for patients with advanced gastric cancer [32]. The Phase III JAVELIN Gastric 300 trial [33] evaluated the efficacy of avelumab in the third-line treatment of patients with advanced gastric cancer. Avelumab was not superior to conventional chemotherapy, according to the results, the median OS was 4.6 months versus 5 months, median PFS was 1.4 months vs. 2.7 months, and ORR was 2.2% vs. 4.3%. However, the incidence of treatment-related adverse events (TRAE) was lower than with chemotherapy (48.9% vs. 74.0%). Immediately thereafter, the phase III JAVELIN Gastric 100 study [34] compared the efficacy of avelumab and chemotherapy as first-line maintenance therapy in patients with HER2-negative gastric cancer, respectively. Whether clearly PD-L1 positive (CPS ≥ 1) or in the overall population, avelumab maintenance therapy did not show significant benefit over continuous chemotherapy in terms of OS but had fewer adverse events than continuous chemotherapy.

### 2.3. Anti-CTLA-4 Antibody

CTLA-4 was the first immune checkpoint identified, and its monoclonal antibody, ipilimumab, was also the first immune checkpoint inhibitor. For gastric cancer, ipilimumab has been used primarily in dual immune therapy in clinical trials. The CheckMate649 study assessed the safety and efficacy of nivolumab in combination with ipilimumab. Due to safety concerns, the duplex group has been discontinued. The available data revealed that the dual combination did not improve overall survival compared with chemotherapy alone in either CPS ≥ 5 or all randomized populations. Nonetheless, combination therapy was more effective in treating MSI-H tumors than chemotherapy alone, with a response rate of 70% compared to 57%. Despite a generally poor response rate, some patients with dual immunotherapy were able to achieve long-term remission. The effectiveness of anti-CTLA-4 in conjunction with PD-1 for gastric cancer may be improved by screening a dominant population.

## 3. Predictive Biomarkers for ICIs

### 3.1. PD-L1

Recent studies have shown a tendency for patients to gain improved therapeutic benefits accompanied by an increased expression of PD-L1. The expression level of PD-L1 has become a favorable indicator for clinical guidance of immunotherapy. Currently, PD-L1 expression in tumor tissue is popularly detected by immunohistochemistry and appraised using the Tumor Proportion Score (TPS) or Combined Positive Score (CPS). The TPS assesses the percentage of PD-L1 positive tumor cells to tumor cells, while CPS evaluates the ratio of PD-L1 positive tumor and immune cells to tumor cells. It has been suggested that CPS is more sensitive than TPS in gastric cancer and, thus, a more widely used [35]. Nevertheless, a comprehensive analysis of 17 randomized controlled trials (RCTs) with minimal risk of bias and 14 forecasting factors by H.H. Yoon et al. [36] revealed that divergent propensities existed in different pathological subtypes. TPS was the most sensitive predictor of whether squamous cell carcinoma patients would benefit from ICI, whereas CPS has the greatest predictive efficacy in adenocarcinoma. The study also validated that PD-L1 was a better predictor of how much gastric cancer patients would benefit from ICI than any other variable other than MSI-H.

Diverse RCTs found disagreements on how to define the CPS values that are definitely advantageous when making predictions. The cohort analysis of the KEYNOTE-059 study indicated that ORR was comparatively higher in the PD-L1 CPS ≥ 1 group than in the CPS < 1 group (15.5% vs. 6.4%) in pembrolizumab-treated patients with previously treated gastric cancer [24]. Pembrolizumab plus chemotherapy was also shown in the KEYNOTE-859 study to have an advantage over chemotherapy monotherapy in patients with CPS ≥ 1, whereas the difference was not statistically significant in those with CPS < 1 [27]. In patients with CPS ≥ 1, 5, and 10, the KEYNOTE-061 trial substantiated that pembrolizumab was superior to paclitaxel with an OS prolonged by 0.8, 1.9, and 2.4 months, respectively [25,37]. This correlation was also confirmed by the Checkmate-649 study [30,38,39]. In either the whole randomized population or patients with a CPS ≥ 5, the trial demonstrated that nivolumab plus chemotherapy improved median OS and PFS in comparison to chemotherapy alone. Nonetheless, the beneficial effect was more pronounced in the subgroup with higher CPS. Notably, the CSCO guidelines for gastric cancer version 2022 incorporated nivolumab plus chemotherapy in class I recommendation for first-line treatment of advanced gastric cancer with CPS ≥ 5 and class II recommendation for first-line treatment of patients with CPS < 5 or inaccessible PD-L1 test, thereby further expanding the application of nivolumab in gastric cancer to the general public.

In addition to the reality that there is not yet a standard cutoff value of PD-L1 expression to guide therapy, variations exiting in the antibodies used for PD-L1 detection. The three primary antibodies currently recognized by the FDA are Ventana SP-142, Dako 22C3, and Dako 28-8 [40]. The companion diagnosis for pembrolizumab and nivolumab is the PD-L1 assay from Dako 22C3 pharmDx and Dako 28-8, respectively. Moreover, the European Commission has authorized Ventana SP263 as a supplemental diagnosis for both immunosuppressive medications [41]. Assay results can vary due to different clinical trials that have employed various antibodies at diverse levels of PD-L1 expression. In the CheckMate-649 trial, for instance, 28-8 was used, and in the KEYNOTE-811 trial, 22C3. Joe Yeong et al. [42,43] used multiplexed immunohistochemistry and immunofluorescence techniques to score PD-L1 CPS, TPS, and immune cells (IC) in 362 gastric cancer samples. Their analysis suggested that compared to the 22C3 and other assays, the 28-8 test can result in a higher proportion of PD-L1 positivity, as well as a relatively high PD-L1 score for PD-L1 assessment. In areas requiring an indication for PD-L1 positivity, the discrepancies between the various tests could have a significant impact on a drug’s eligibility.

### 3.2. Mismatch Repair Deficiency/Microsatellite Instability (dMRR/MSI)

Microsatellite unstable (MSI) tumors, Epstein-Barr virus-positive tumors, genomically stable(GS), and tumors exhibiting chromosomal instability (CIN) are the four subtypes divided by the Cancer Genome Atlas (TCGA) research network [44]. The MSI of tumors, which is frequently brought on by mutations in the mismatch repair (MMR) gene and functional defects, is a phenomenon in which the length of microsatellite sequences is altered by insertion or deletion mutations during DNA replication [45,46].

In advanced gastric cancer, the genotype for mismatch repair deficiency (dMRR) accompanied by MSI high status presents in nearly 6% of patients [47]. The dMRR/MSI-H tumors have been deemed to achieve therapeutic advantage for benefiting from anti-PD-1(L1) therapy because they were indicated to show a hypermutated phenotype with increased tumor-specific neoantigens and rising frequency of tumor-infiltrating lymphocytes (TILs), particularly CD8+ TILs [48,49].

Anti-PD-1 therapy has been significantly beneficial for treating dMMR/MSI-H solid tumors, according to several clinical trials. The ORR of pembrolizumab in the KEYNOTE-059 study was 57.1% in patients with MSI-H (≥ 20 mutations/MB) gastric cancer compared to 9.0% in individuals with no MSI-H [24]. Additionally, KEYNOTE-061 showed that patients with MSI-H gastric cancer responded better to pembrolizumab monotherapy than to paclitaxel monotherapy [37]. PD-1 monoclonal antibody monotherapy also outperformed its combination with chemotherapy in the first-line treatment of MSI-H gastric cancer, according to a subgroup analysis of KEYNOTE-062. In patients with resected primary gastric cancer, MSI is independently associated with DFS and OS and is a reliable prognostic indicator [50]. According to published data from Checkmate-649, the median OS was significantly better (38.7 vs. 12.3 months) in the nivolumab plus chemotherapy group than in the chemotherapy monotherapy group for unique patients with MSI-H advanced gastric cancer. Additionally, compared to the chemotherapy group, the combined group demonstrated a superior ORR (55% vs. 39%) and a 62% decreased risk of death. A meta-analysis of several randomized trials of immunotherapy for gastric cancer with published data, including KEYNOTE-061, KEYNOTE-062, CheckMate649, and JAVELIN in gastric cancer 100, was undertaken by F. Pietrantonio et al. [51]. The prognosis of the dMMR/MSI-H advanced gastric cancer patient population was dramatically improved by immunotherapy alone or in combination with immunotherapy, according to the statistics, compared to chemotherapy alone.

Based on sound medical evidence, the FDA granted nivolumab and pembrolizumab for second or third-line treatment in all individuals with dMMR/MSI-H solid tumors [52]. The CSCO 2022 gastric cancer guideline adopts dMMR/MSI-H as a routine test for all patients with newly diagnosed gastric cancer regardless of HER-2 status and, moreover, offers new treatment recommendations for the dMMR/MSI-H population. Previously, dMMR/MSI-H was solely mentioned as a suggested testing element. Regardless of HER2 status, pembrolizumab monotherapy is recommended as a Class II first-line therapy for dMRR/MSI-H advanced metastatic gastric cancer based on the precise long-term survival benefit. Furthermore, a class III first-line therapy combining nivolumab and ipilimumab is advised. For patients with advanced metastatic gastric cancer who are receiving the proper treatment, the dMMR/MSI-H status emerges as a novel classification factor [13].

### 3.3. Tumor Mutation Burden (TMB)

TMB is known as the overall number of substitutions, insertions, and deletions mutation per megabase in the gene exon’s coding area that has been determined in a tumor sample. The TMB varies considerably among cancer types, with the median TMB in gastric cancer being 3.69 mutations/Mb [53]. Tumor cells with elevated genetic mutations are more likely to synthesize aberrant proteins that raise the likelihood that the immune system will recognize them, triggering an immunological response that makes immunotherapy feasible for patients [54,55]. Alexandra Snyder et al. [56] identified an association between mutational load and the degree of therapeutic benefit in patients by analyzing the genetic basis of CTLA-4 blocker therapy in melanoma patients. Following this, Rizvi et al. [57] underwent whole-exome sequencing on non-small cell lung cancer patients receiving pembrolizumab and discovered a similar link between a higher burden of neoantigens or nonsynonymous mutations and better outcomes for the treatment of 27 cancers with ICIs, Yarchoan et al. [58] collected data from numerous clinical studies to depict the linearity between median TMB and ORR. They identified a significant positive correlation between TMB and ORR (*p* < 0.001) with a correlation coefficient of 0.74.

One thousand and seventy-three patients with advanced solid tumors were enrolled in the phase II KEYNOTE-158 study, 76% of whom were eligible for TMB status evaluation [59]. Patients with tumor mutation burden-high (TMB-H) had a significantly higher ORR (29% vs. 6%) than other patients when using a threshold of 10 muts/Mb. Accordingly, the National Comprehensive Cancer Network (NCCN) guideline recommended incorporating pembrolizumab monotherapy into second-line and later for advanced solid tumors with TMB ≥ 10 muts/Mb [14]. However, the efficacy of KEYNOTE-158 may not be representative of all types of tumors due to some common cancer types (such as colorectal cancer and gastric cancer) being left out of the prospective biomarker analysis in this trial. The correlation between TMB-H and ICI benefit for gastric cancer has been partially confirmed; for instance, the KEYNOTE-061 study previously validated that gastric cancer patients with high TMB received pembrolizumab with significantly better PFS and OS than paclitaxel monotherapy. The effectiveness of pembrolizumab combined with chemotherapy in the first-line treatment of advanced gastric cancer was also assessed as part of the KEYNOTE-062 research [60]. Patients with TMB ≥ 10 muts/Mb obtained a better survival benefit of OS, PFS, and ORR. Remarkably, 44% of patients with TMB ≥ 10 muts/Mb exhibited MSI-H concurrently, and by excluding this subset of patients, the association between TMB and efficacy would be diminished. The median OS of TMB-H (≥12 muts/Mb) patients was 14.6 months versus 4.0 months (*p* = 0.038) from tumor mutation burden-low (TMB-L) (<12 muts/Mb) patients with an ORR of 33.3% versus 7.1% (*p* = 0.017) in a phase II study of toripalimab for the treatment of refractory gastric cancer [61]. Some cut-off values of TMB in advanced gastric cancer studies are listed in Table 2.

Nevertheless, the proof that TMB-H predicts the performance of immunotherapy for gastric cancer is insufficient, according to an analysis of pan-solid tumors with 1661 individuals by McGrail et al. [62], TMB-H had no predictive value for gastric cancer but retained its predictive value for NSCLC, head and neck squamous cell carcinoma, and melanoma, etc. Moreover, one study has demonstrated that TMB status does not imply an overall remission rate for gastric cancer of the MSS subtype. In a retrospective study, Wang et al. [63] examined the viability of TMB as a predictive biomarker of ICIs antitumor effect in MSS gastrointestinal tumors. The prevalence of TMB-H was only 3.29% in MSS gastrointestinal tumors. To date, there is insufficient evidence that MSS gastrointestinal tumors with TMB of 10 muts/Mb or higher can observably benefit from ICIs therapy. Hence, TMB-H is a biomarker with predictive value but is still predominantly challenging in gastric cancer.

### 3.4. EBV-Positive Status

As mentioned above, an EBV-positive tumor is one of the four subtypes in the TCGA classification. In gastric cancer, Epstein–Barr Virus is considered a carcinogen detected by 8–10% of patients [64,65]. A patient with advanced metastatic gastric cancer was found to show clinical benefit in the treatment of avelumab by Panda, A. et al. [66]. They then performed a statistical analysis of the TCGA database and found that EBV-positive tumors possessed lower mutational load than MSI tumors but reversely showed more obvious evidence of immune infiltration. In addition, upward expression of immune checkpoint pathways (PD-1, CTLA-4) at the RNA level and elevated histologic lymphocyte infiltration were simultaneously observed in EBV-positive tumors over MSS tumors. Given the strong correlation between immunity and EBV-positive tumors, EBV status may be a predictive biomarker for precision medicine in the management of gastric cancer [67].

Even so, more investigation is required to figure out exactly how EBV infection determines the potency of ICI against gastric cancer and the underlying mechanisms. Pembrolizumab was prescribed to treat six cases of metastatic gastric cancer that was EBV positive in Korea, and the authors Kim, S.T. et al. [68] reported an impressive ORR of 100%. However, Wang et al. [61] reported that only one individual achieved partial remission (PR) in four patients with EBV-positive gastric cancer, whereas two cases of stable disease and one case of disease progression. Undesirably, the one who went into PR possessed a positive PD-L1 expression, while negative in the other three patients.

EBV-encoding RNA in situ hybridization (ISH) has long been the gold standard for EBV detection. By evaluating the transcriptional status of seven key EBV genes, J. Yuan et al. [69] was able to determine the EBV expression status in gastric cancer at the RNA level. This study established an EBV RNA-based NGS panel with seven genes involved, containing EBER1, EBER2, EBNA1, and BZLF1, BARF1, LMP1, and LMP2A/B, to assess EBV infection status. The analysis revealed the characteristic that a significantly higher expression profile of EBER1/EBER2 exists in EBV-positive samples, which points to the potential utility of an RNA-based NGS panel to identify the presence of EBV in gastric cancer. The RNA-based NGS panel is a promising alternative to the single ISH approach for determining EBV status in gastric cancer.

### 3.5. Tumor Immune Microenvironment (TIME)

TIME refers primarily to immune cells and associated immune molecules in the tumor microenvironment, which underlie tumor development and metastasis [70,71]. Many studies have shown that the efficacy of immune checkpoint inhibitors is directly related to TIME [72]. Tumor-infiltrating lymphocytes (TILs), consisting of T cells, B cells, and NK cells, can infiltrate tumor cells and the surrounding stroma [73,74]. According to a clinical trial, substantial TILs infiltration in gastric cancer tissue was linked to a good prognosis and may be a reliable predictor of protective factors. Patients with high TIL levels outlived patients with low TIL levels in terms of both overall survival and progression-free survival, and TILs in tumor mesenchyme may assist in representing the biological activities of various Th subpopulations in the tumor immunity [75]. Furthermore, Boku et al. proved that myeloid cells, a crucial part of the TIME, can express a number of immune checkpoint molecules, such as PD-1, CTLA-4, and LAG3, and can facilitate tumor metastasis. Then, in the WJOG10417GTR study, they examined TILs and PBCs in 91 patients with advanced gastric cancer both before and after nivolumab monotherapy. The group with a high proportion of CTLA-4 and LAG3+ myeloid cells before nivolumab treatment had significantly shorter PFS and OS, as well as a poor response to ICI [76,77,78]. Patients with HER2-negative gastroesophageal cancer in the phase II PLATFORM study who received durvalumab as maintenance therapy after undergoing platinum-fluorouracil as first-line therapy did not have a prolonged OS or PFS. Based on the immunological and angiogenic axis, the Xerna^TM^ TME RNA group divides TME into Immune Active (IA), Immune Suppressed (IS), Angiogenic(A), and Immune Desert (ID) phenotypes. Patients were all pMRR, with a high immune score of 51.2% (IA+IS) and a PD-L1 ≥CPS of 54.9%. Patients with high immune scores (IA+IS) had markedly improved PFS at 6 and 12 months and a superior OS at 24 months than patients with low immune scores (A+ID). Additionally, compared to patients with CPS < 5, those with CPS ≥ 5 got better PFS at 12 months only but neither PFS at 6 months nor OS at 24 months. In comparison to PD-L1 CPS ≥ 5, the researchers contended that the high immune score phenotype (IA+IS) might profit from durvalumab maintenance medication and more accurately identify individuals who will benefit from ICI. Additionally, based on the spatial arrangement of CD8+PD-1+LAG3- T cells and the density of CD8+PD-1-LAG3-, CD68+STING+, and CD4+FoxP3-PD-L1+ cells, a multi-dimensional marker of tumor-infiltrating immune cells (TIICs) has been successfully developed to predict the response to immunotherapy [79]. Again, it was verified that TIICs play an essential role in predicting the immune response.

### 3.6. Specific Genetic Mutations

The DNA mismatch repair mechanism and the DNA polymerase correction mechanism are the two fundamental systems that guarantee the precision of the genome replication [80]. POLD1 and PLOE, which encodes the proofreading and catalytic subunits of DNA polymerase δ and polymerase ε, respectively, are crucial for DNA replication and the proofreading [81]. Loss of proofreading ability brought on by POLE/POLD1 gene mutations permits the accumulation of mutant genes in cells. Neoantigens with functional mutations in POLE/POLD1 show increased hydrophobicity of the TCR-contacting residues than their wild-type counterparts, enhancing their recognition by and capacity to activate T cells [82]. Emerging studies have demonstrated that POLE/POLD1 mutations are related to ICI efficacy and are predicted to be the next independent biomarker for predicting immunotherapy response [83,84,85]. By examining the mutation data from 47,721 individuals with cancers, Feng Wang et al. [86] identified that POLE/POLD1 gene mutations were seen in a number of gastrointestinal tumors and were present in about 7% of patients with gastroesophageal cancer. The median OS of patients with POLE/POLD1 mutant tumors was noticeably longer than wild-type patients (34 vs. 18 months), and 74% of them had a status of MSS or MSI-L. POLE/POLD1 mutant patients also outlived wild-type patients by a large margin in non-MSI-H patients (28 vs. 16 months).

Mutations of NOTCH4 have also recently been proven to be predictive of ICI efficacy [87]. As a member of the NOTCH family, NOTCH4 has been amply demonstrated to participate in tumor invasion, differentiation, proliferation, and apoptosis in a spectrum of different tumor cells [88,89]. One study [90] pooled published genomic and efficacy data from seven studies of patients treated with ICI alone or in combination, including 40 cases of gastroesophageal cancer. The exploration cohort showed an ORR of 42.9% for immunotherapy in NOTCH4 mutants, considerably higher than the 25.9% for the wild type (*p* = 0.007). In comparison to the wild type, mutant patients exhibited a sizable advantage in terms of long-lasting clinical benefits, PFS, and OS. The researchers found a substantial correlation between NOTCH4 mutations and enhanced immunogenicity, including TMB, co-stimulatory molecule overexpression, and activation of antigen processing pathways. Moreover, immunological responses against the tumor, such as immune cell infiltration and various immune markers, were favorably correlated with the mutations.

### 3.7. Gut Microbiota

Several studies have revealed that gut microbiota can potentially have an impact on the efficacy of ICIs. For the first time, mycobacteria from mice and melanoma patients were shown to play a pivotal role in immune stimulation of CTLA-4 blockade, with the antitumor effects of CTLA-4 blockade dependent on different mycobacterial species [91]. In parallel, another study demonstrated differential spontaneous tumor immune effects in melanoma mice with different commensal flora and confirmed the association of Bifidobacterium with antitumor effects [92]. This has prompted oncologists to consider the relationship between gut microbiota and antitumor immunity. Evidence has shown that the gastroesophageal microbiota plays an influential role in programming innate and adaptive immunity. The DELIVER [93] study aims to explore whether genomic information about the gut flora can predict the efficacy of nivolumab in advanced gastric cancer. The primary endpoint of the study was the relationship between genomic pathways in the gut flora group and nivolumab, independent of disease progression at the time of the first evaluation. Upregulation of the bacterial invasive epithelial cell pathway (KEGG pathway) was associated with the presence of PD at the first assessment after nivolumab treatment (training cohort: *p* = 0.057; validation cohort: *p* = 0.014). Furthermore, the microbiome was more diverse in non-PD patients than in PD patients. Analysis of bacterial species revealed that *Veillonella* and *Odoribacter* species were associated with the efficacy of nivolumab treatment. Gut flora pathways would be expected to predict the efficacy of ICIs in advanced gastric cancer.

### 3.8. Liquid Biopsy Biomarkers

The majority of biomarkers above are identified primarily through pathological biopsy, an invasive procedure that hurts patients. Compared to tissue biopsy, liquid biopsy is less invasive and can dynamically follow tumor variation in patients in real-time. The significance of liquid biopsy is to examine certain markers such as exosomes, circulating tumor cells (CTC), or circulating tumor DNA (ctDNA) [94]. It has been discovered that ctDNA, the DNA of tumor cells entering the circulatory system after elimination or apoptosis, can serve as a distinctive biomarker. The effectiveness of immunotherapy for a broad spectrum of cancers can be forecasted by alterations in the genomic instability of the ctDNA [95]. In 46 patients receiving PD-1 monoclonal antibody, Jin Y et al. [96] performed 425-genes NGS testing. In comparison to individuals without a drop, those with a decrease in maximal somatic variant allelic frequency (maxVAF) of more than 25% exhibited a longer PFS (7.3 vs. 3.6 months) and a greater rate of treatment response (53.3% vs. 13.3%). Patients with FGFR4, CEBPA, KMT2B, or MET variations had a higher risk of immune-related adverse events (*p* = 0.09), while RHOA, TGFBR2, and PREX2 mutational status affected PFS with immunotherapy (p0.05). Additionally, patients treated with pembrolizumab and trastuzumab induction therapy exhibited an association between ctDNA decline and improved survival in the KEYNOTE 811 trial [97]. Median PFS was 14.7 (11.0-NR) months for patients with decreased ctDNA compared with 5.9 (4.1-NR) months for those with elevated ctDNA. Median OS was 29.7 (27.2-NR) vs. 7.71 (6.6-NR) in parallel. ctDNA clearance at the 9th week predicted improved PFS (median PFS: 12.4 vs. 2.9 months). Additionally, exosomes are adaptable, cell-derived nanovesicles with the capacity to penetrate bodily barriers and exact targeting specificity. Due to their great stability, exosomes are more abundant in the bloodstream than sources like CTC and ctDNA, making them a better choice for liquid biopsy [98]. Circulating exosomal PD-L1 has been shown in a study by Chen G et al. to be a reliable indicator of response to anti-PD-1 therapy in the melanomas [99]. Zhang H et al. [100] demonstrated that exosomes secreted by gastric cancer cells carry and translocate EGFR to the liver, where EGFR evolves the liver microenvironment and, thus, promotes successful colonization of the liver by metastatic cancer cells. Furthermore, exosomal PD-L1 was found to be a standalone prognostic factor in GC by Yibo Fan et al. When compared to the low exosomal PD-L1 group, the OS was considerably lower in the high exosomal PD-L1 group. Meanwhile, CD4+ T cell count, CD8+ T cell count, and granzyme B were inversely correlated with exosomal PD-L1 in the plasma samples of 31 patients with metastatic GC, demonstrating that exosomal PD-L1 was related to immunosuppressive status in GC patients [101]. The results of these researches indicated the potential of circulating exosomes as predictors of immune efficacy.

### 3.9. Comprehensive Predictive Effect of Biomarkers

Although multiple predictors have been identified, any single predictive biomarker has its own limitations and cannot reliably recognize the recipient population. It may be possible to increase prediction sensitivity and accurately access the immunological status of individuals through the application of integrated detection or the establishment of reliable predictive models. Gjoerup et al. [102] examined the link between PD-L1, TMB, and MSI in 22,592 patients with various malignancies and discovered a significant percentage of patients possessed more than two positive biomarkers, highlighting the value of combination testing in clinical treatments. The prevalence of PD-L1 positivity, TMB-H, and MSI-H amplification varies widely among tumor types, and when these markers are combined, they serve as a more efficient screening tool for immunotherapy potential. The range of PFS and OS overlapped between the different PD-L1 expression subgroups and Xie et al. [103] found that the correlation between PD-L1 expression and ORR was not obvious during immunotherapy combined with chemotherapy, indicating that PD-L1 expression alone is not the sole factor guiding combination immunotherapy in gastric cancer. Additionally, in the future, if machine learning and artificial intelligence can be utilized to build multivariate models to predict the efficacy of immunotherapy using data from large samples of the tumor and its microenvironment, it will aid in the development of a new paradigm for precision tumor therapy.

## 4. Conclusions

The development of gastric cancer is multi-factorial, and the average gene copy number varies much more than for other tumor types, indicating that gastric cancer is a very heterogeneous tumor. In recent years, great advances have been achieved in immunotherapy modalities, providing more options for patients with advanced metastatic gastric cancer. Unfortunately, only some patients respond to ICIs therapy. The issues of predicting immune efficacy and precisely selecting the population for therapeutic benefit have been raised. PD-L1, dMRR/MSI-H, and TMB have been validated in some clinical trials to predict immune response to ICIs and are relatively mature immune markers. Emerging biomarkers, such as driver mutations, tumor-infiltrating lymphocytes, liquid biopsy biomarkers, and gut microbes, which are still being explored, also serve as potential predictors of the efficacy of ICIs in gastric cancer in the future. However, there are currently many gaps in the use of these markers in clinical studies. For markers whose measurements are continuous variables, such as TMB and PD-L1, the way to classify the risk threshold has not been standardized. Some biomarkers (PD-L1) are heterogeneous in time or space, possibly reducing the accuracy of prediction for individuals. In addition, most clinical trials do not involve enough treatment samples and inconsistent study methods, which need to be validated by high-quality standardized clinical studies for individualized and precise treatment of gastric cancer. Given the complexity of the immune system and its regulatory mechanisms, a single biomarker cannot predict the efficacy of immunotherapy for all patients or for all types of immunotherapy. In the future, multidimensional or dynamic marker assays could be a possible way forward.

## Figures and Tables

**Table 1:** Clinical trials of anti-PD-1 antibody for GC.

Clinical Trial	Reference	Country	Line	Stage	CPS	Study Design	mOS (Month)	mPFS (Month)	ORR (%)
ATRATION-2	[21]	Japan, South Korea, Taiwan	Third or later	III	all	Nivolumab	5.3	-	-
						Placebo	4.1	-	-
ATRATION-4	[22]	Japan, South Korea, Taiwan	first	III	all	Nivolumab + chemo	17.5	10.5	-
						Placebo + chemo	17.2	8.3	-
CheckMate-649	[23]	29 countries	first	III	CPS ≥ 5	Nivolumabm + chemo	14.4	8.3	60
						chemo	11.1	6.1	45
KEYNOTE-059	[24]	16 countries	Third or later	II	CPS ≥ 1	pembrolizumab	-	-	15.5
					CPS < 1		-	-	6.4%
KEYNOTE-061	[25]	30 countries	second	III	CPS ≥ 1	Pembrolizumab	9.1	1.5	-
						Paclitaxel	8.3	4.1	-
KEYNOTE-062	[26]	29 countries	first	III	CPS ≥ 1	Pembrolizumab	10.6	2.0	14.80
						Pembrolizumab + chemo	12.1	6.9	48.60
						placebo + chemo	11.1	6.4	37.20
KEYNOTE-859	[27]	31 countries	first	III	CPS ≥ 1	Pembrolizumab + chemo	12.9	6.9	51.3
						placebo + chemo	11.5	5.6	42
KEYNOTE-811	[28]	20 countries	first	III	CPS ≥ 1	Pembrolizumab + trastuzumab + c hemo	-	-	74.4
						Placebo + trastuzumab + chemo	-	-	51.9
ORIENT-16	[29]	China	first	III	CPS ≥ 5	Sintilimab + chemo	18.4	7.7	-
						Placebo + chemo	12.9	5.8	-

CPS: Combine Positive Score; mOS, median overall survival; mPFS, median progression-free survival; ORR, objective response rate; chemo, chemotherapy.

**Table 2:** The cut-off value of TMB in advanced gastric cancer.

Clinical Trial	Detection Method	Numbers of Patients	Cut-Off Value	TMB-H (%)
NCT0291543	NK	54	≥12 muts/Mb	22%
KEYNOTE-601	WES	420	≥175 muts/Exons	18%
KEYNOTE-601	FoudationOneCDx	204	≥10 muts/Mb	17%
KEYNOTE-602	FoudationOneCDx	306	≥10 muts/Mb	16%

WES, whole-exome sequencing.
